# *Helicobacter pylori* Outer Membrane Vesicles Protect the Pathogen From Reactive Oxygen Species of the Respiratory Burst

**DOI:** 10.3389/fmicb.2018.01837

**Published:** 2018-09-07

**Authors:** Sujinna Lekmeechai, Yu-Ching Su, Marta Brant, Maria Alvarado-Kristensson, Anna Vallström, Ikenna Obi, Anna Arnqvist, Kristian Riesbeck

**Affiliations:** ^1^Clinical Microbiology and Molecular Pathology, Department of Translational Medicine, Faculty of Medicine, Lund University, Malmö, Sweden; ^2^Department of Medical Biochemistry and Biophysics, Umeå University, Umeå, Sweden

**Keywords:** *H. pylori*, KatA, outer membrane vesicles, oxidative burst, reactive oxygen species

## Abstract

Outer membrane vesicles (OMVs) play an important role in the persistence of *Helicobacter pylori* infection. *Helicobacter* OMVs carry a plethora of virulence factors, including catalase (KatA), an antioxidant enzyme that counteracts the host respiratory burst. We found KatA to be enriched and surface-associated in OMVs compared to bacterial cells. This conferred OMV-dependent KatA activity resulting in neutralization of H_2_O_2_ and NaClO, and rescue of surrounding bacteria from oxidative damage. The antioxidant activity of OMVs was abolished by deletion of KatA. In conclusion, enrichment of antioxidative KatA in OMVs is highly important for efficient immune evasion.

## Introduction

*Helicobacter pylori* is a Gram-negative pathogen that commonly colonizes the gastric mucosa. Infection persists for a lifetime without antibiotic treatment although the pathogen constantly experiences hostile conditions including the acidic ventricle environment and host defense (Roesler et al., 2014). In order to survive against the highly acidic gastric juice (pH 1.0–3.0), *H. pylori* uses a series of acidic acclimation systems that neutralize the surrounding acid. Other virulence mechanisms include expression of abundant molecules at the surface for attachment and manipulation of host extracellular matrix proteins and serum resistance (Parker and Keenan, 2012; Richter et al., 2016). In addition, *H. pylori* is equipped with antioxidant molecules such as catalase (KatA), catalase-like protein (KatB), alkyl hydroperoxide reductase (AhpC), and superoxide dismutase (SOD) to detoxify reactive oxygen species (ROS) released from host immune cells during the respiratory burst (Wang et al., 2006). Furthermore, *H. pylori* constitutively releases outer membrane vesicles (OMVs) from its outer membrane (OM).

Outer membrane vesicles are cargos comprising an OM lipid bilayer enveloping several virulence factors. *H. pylori* OMVs have been extensively studied with respect to composition, proteome, and virulence functions (Mullaney et al., 2009; Olofsson et al., 2010), and play multiple roles in bacterial pathogenesis including biofilm formation, cancer development, and immune evasion (Parker and Keenan, 2012). Furthermore, OMVs display immunomudulatory effects by inducing IL-8 secretion from epithelial cells, activating phagoctyes, and suppressing immune cells of the adaptive immune system (Mullaney et al., 2009; Olofsson et al., 2010; Ko et al., 2016).

KatA, a 55 kDa catalase, is an essential virulence factor protecting *H. pylori* against the respiratory burst (Olofsson et al., 2010). In fact, KatA is upregulated during oxidative stress (Huang and Chiou, 2011). It is widely known that KatA detoxifies hydrogen peroxide (H_2_O_2_) and hypochlorite (OCl^-^) (Benoit and Maier, 2016). Additionally, we recently reported that KatA mediates vitronectin acquisition resulting in increased serum resistance (Richter et al., 2016). Interestingly, despite the lack of a signal peptide, *Helicobacter* KatA is ubiquitous with various topology including the bacterial surface, the cytosol and periplasmic space. KatA has also recently been identified in OMVs (Wang et al., 2006; Mullaney et al., 2009). However, little is known regarding the role of KatA in OMVs since previous studies have mainly focused on KatA in the cell-associated context.

We determined the importance of OMVs to eliminate extracellular ROS-mediated killing via KatA enrichment. Our data suggest a new mechanism of OMV-mediated *H. pylori* evasion from the attack of the innate immune system.

## Results

### KatA Catalase Is Enriched in *H. pylori-*Derived OMVs

**FIGURE 1:**
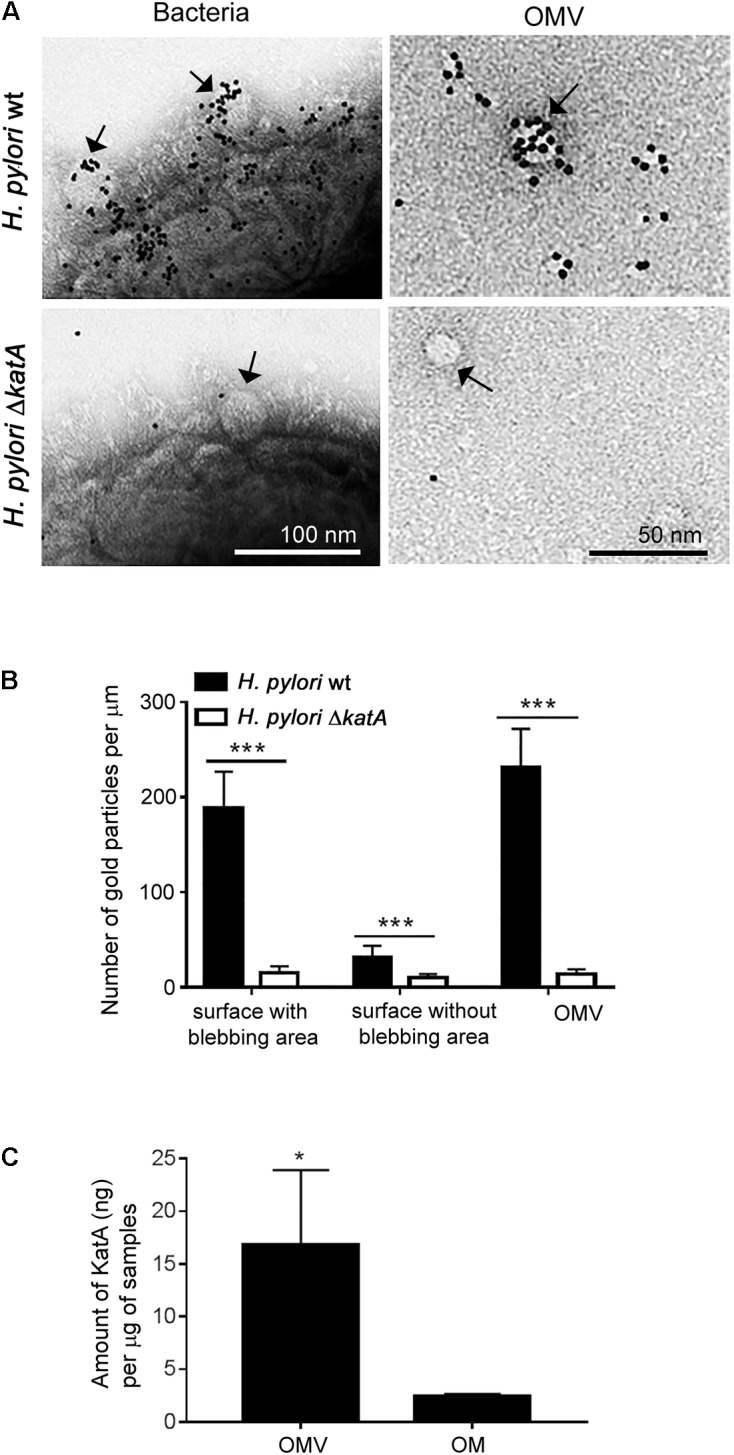
Characterization of KatA enrichment and deposition at the surface of *Helicobacter* outer membrane vesicles (OMVs). **(A)** Detection of KatA at the surface of OMVs by immunoelectron microscopy using gold conjugated rabbit anti-KatA IgG pAb (black particles) at the outer surface of intact *H. pylori* 18943 wt (upper panel) or at the outer surface of the KatA-deficient mutant *H. pylori* 18943Δ*katA* (lower panel). Arrows indicate OMVs that are vesiculating from the “blebbing area” at the bacterial surface (left panels) or purified OMVs (right panels). Visualization by Transmission Electron Microscopy (TEM) was performed on a Philips/FEICM 100 TWIN transmission electron microscope, and images were documented with a side-mounted Olympus Veleta camera having a resolution of 2048 × 2048 pixels (2k × 2K) and ITEM acquisitions software. **(B)** KatA is accumulated in OMVs at the bacterial surface, and in the released OMV fraction. The number of anti-KatA IgG pAb-gold particles per μm from 50 randomly selected TEM image profiles were calculated, and corresponded to at least 1000 different bacteria. **(C)** A significantly higher concentration of KatA is present in OMVs compared to the OM fraction. Estimation of KatA concentrations in OM and OMVs was done by western blotting as shown in **Supplementary Figure S1**. For **(B)** and **(C)**, statistical differences were calculated by two-way ANOVA and two-tailed Student’s *t*-test, respectively (mean ± SD; *n* = 3; ^∗^*p* < 0.05; ^∗∗∗^*p* < 0.001).

*Helicobacter pylori* KatA has been predicted as one of the periplasmic proteins that accounts for 7.4% of the total OMV proteome (Mullaney et al., 2009; Olofsson et al., 2010). Since most OM proteins are also located at the surface of vesicles (Bonnington and Kuehn, 2014), we wanted to investigate whether KatA localizes at the outer surface of *H. pylori* OMVs. As visualized by TEM, we found deposition of gold-labeled anti-KatA pAb at the surface of intact bacteria and OMVs of *H. pylori* wild type (wt) (**Figure 1A**). However, no KatA was detected on any samples derived from the KatA-deficient *H. pylori* Δ*katA* mutant. This suggested a similar surface exposure of KatA on OMVs as seen on intact bacteria. Further enumeration of antibody deposition revealed that more KatA was detected at the “blebbing areas” of wild type bacteria as compared to the “non-blebbing areas,” and this appearance was almost similar to the OMVs (**Figure 1B**). This observation prompted our interest to compare the amount of KatA present in the OMVs and OM of *H. pylori*. Interestingly, we observed that OMVs contained sevenfold more KatA (18.37 ± 6.24 ng/μg sample) than bacterial OM (2.42 ± 0.24 ng/μg sample) (**Figure 1C** and **Supplementary Figure S1**).

### KatA Enriched OMVs Exhibit Catalase Activity

**FIGURE 2:**
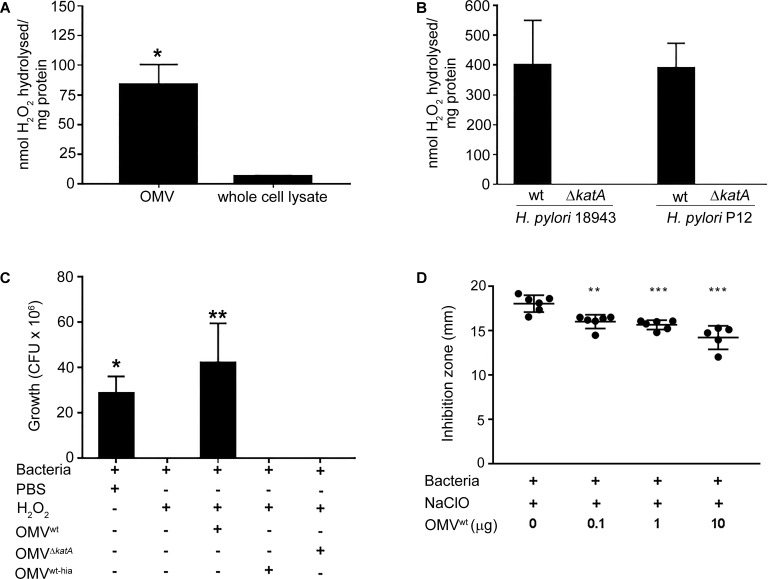
OMVs carrying KatA hydrolyse/detoxify ROS and provide protection against ROS-mediated bacterial killing. **(A)** Comparison of catalase activity between OMVs derived from *H. pylori* 18943 wt and a bacterial lysate. An increased H_2_O_2_ hydrolysis on a weight basis was detected with OMVs compared to bacterial lysate. **(B)** Determination of KatA-dependent catalase activity in OMVs. Deletion of KatA in two different strains of *H. pylori*, 18943 and P12 resulted in abolished catalase activity in OMVs from *H. pylori*Δ*katA* mutant strains compared to OMVs derived from the wild type counterparts. In **(A)** and **(B)**, catalase activity was presented as nmol of H_2_O_2_ decomposed per mg of sample tested (mean ± SD; *n* = 3*;* ^∗^*p* < 0.05; ^∗∗^*p* < 0.01). **(C)** and **(D)**, *Helicobacter* OMVs reduce the bactericidal activity of H_2_O_2_ and NaClO, and thus promoted bacterial survival. In **(C)**, *H. pylori* P12Δ*katA* devoid of catalase activity was challenged with 1 mM H_2_O_2_ preincubated with 40 μg/ml of OMV (equivalent to OMVs produced from 10^9^ CFU) (OMV^wt^, OMV^Δ^*^katA^* or heat-inactivated OMVs; OMV^hia^). In **(D)**, *H. pylori* 18943*ΔkatA* devoid of KatA expression was exposed to 5% NaOCl that had been pre-treated with 0.1–10.0 μg of OMV^wt^. Pure H_2_O_2_ and NaOCl in PBS that were fully bactericidal were included as a positive control. In **(C)**, the viability of bacteria was assessed by plating and counting colony forming units (CFU). Only KatA-containing OMV^wt^ was able to neutralize H_2_O_2_ and promote survival of *H. pylori* P12Δ*katA* from peroxidative killing. OMV lacking KatA (OMV^Δ^*^katA^*) or heat-inactived KatA (OMV^hia^) did not show any protection of whole bacteria (mean ± SD; *n* = 3*;* ^∗^*p* < 0.05; ^∗∗^*p* < 0.01). In **(D)**, the bactericidal activity of NaOCl was decreased by preincubation with increasing amounts of OMV^wt^ (equivalent to OMVs produced from 0.2 to 2 × 10^8^ CFU). The killing of *H. pylori* 18943Δ*katA* by NaOCl was measured by the diameter of inhibition zone, which is the area without bacterial growth (mean ± SD; *n* = 3*;* ^∗∗^*p* < 0.01; ^∗∗∗^*p* < 0.001).

*Helicobacter* KatA of intact bacteria is known to actively hydrolyse H_2_O_2_ and detoxify ClO^-^ (Wang et al., 2006; Benoit and Maier, 2016). Interestingly, we found that the H_2_O_2_ hydrolysis activity in OMVs was significantly (*p* < 0.05) higher than the *H. pylori* wt whole cell lysate (**Figure 2A**). We further investigated whether KatA could contribute to the antioxidant activity of *Helicobacter* OMV. As shown in **Figure 2B**, OMVs isolated from the strain *H. pylori* 18943 wt exhibited a strong catalase activity based upon hydrolysis of H_2_O_2_ compared to OMVs of the *H. pylori* 18943Δ*katA* mutant that had an abolished KatA activity. In parallel, a similar H_2_O_2_ hydrolysis activity was also found with OMVs isolated from another *H. pylori* strain (P12), whereas no activity was observed with the corresponding KatA-deficient *H. pylori* P12 mutant.

### KatA-Enriched OMVs Promote *H. pylori* Survival Against the ROS of the Oxidative Burst

We also wanted to determine whether OMV loaded with KatA could protect bacteria from the bactericidal activity of ROS (H_2_O_2_ and ClO^-^) (Wang et al., 2006; Benoit and Maier, 2016). First, *H. pylori* Δ*katA* lacking the catalase activity was exposed to H_2_O_2_ that had been pre-incubated with OMVs derived from *H. pylori* wt (OMV^wt^) or the KatA-deficient mutant (OMV^Δ^*^katA^*). Interestingly, *H. pylori* Δ*katA* survived when OMV^wt^-pre-treated H_2_O_2_ was added, but was completely killed in both H_2_O_2_ or H_2_O_2_ preincubated with OMV^Δ^*^katA^* (**Figure 2C**). Since KatA activity is heat sensitive, OMV^wt^ was also heat-inactivated at 60°C to generate OMV^wt-hia^. We found that H_2_O_2_ preincubated with OMV^wt-hia^ remained bactericidal against the mutant *H. pylori* Δ*katA.*

We subsequently performed a disk diffusion assay to examine the capacity of OMVs in protecting *H. pylori* from the toxicity of NaClO. As shown in **Figure 2D**, the inhibition zone of *H. pylori* Δ*katA* growth caused by NaClO was gradually reduced in response to increasing amounts (0.1–10 μg) of OMV^wt^ used for preincubation with NaClO. Taken together, our data indicated that *H. pylori* OMVs exhibiting KatA-dependent catalase activity successfully neutralized both H_2_O_2_ and NaClO, and thus promoting bacterial survival when exposed to the bactericidal activity of ROS.

## Discussion

*Helicobacter pylori* has evolved several virulence mechanisms for persistent colonization and infection in the gastric mucosa and this includes release of OMVs (Parker and Keenan, 2012). Here, we deciphered a novel role of OMVs in the pathogenesis of *H. pylori*; OMVs act as antioxidative particles via enrichment of KatA at the surface of vesicles. To the best of our knowledge, the current study is the first report regarding enrichment of KatA in *H. pylori* OMVs. Intriguingly, the *H. pylori* virulence factors OipA and HtrA have also been reported to be enriched in OMVs (Olofsson et al., 2010).

Production of toxic ROS, *i.e.*, superoxide (O_2_^∙-^), nitrogen oxide (NO), H_2_O_2_, and OCl^-^ by human polymorphonuclear cells (PMNs) during the respiratory burst is an important component of the innate defense to eradicate phagocytosed pathogens (Yang et al., 2013). Despite *H. pylori* infection induces massive influx of neutrophils into the gastric mucosa and production of ROS, the pathogen expresses KatA to survive at the surface of phagocytes, *H. pylori* is thus antiphagocytic and resistant against the respiratory burst-dependent killing (Ramarao et al., 2000; Wang et al., 2006). In contrast to bacterial cell-associated KatA, little is known regarding the KatA-dependent ROS resistance of OMVs.

We speculated that the accumulation of KatA in *H. pylori* OMVs, and thus higher catalase activity compared to bacterial cells (**Figure 2A**), may confer OMVs as an antioxidant cargo to protect bacteria from extracellular ROS of the respiratory burst. Bacterial interactions with PMNs result in an increase of extracellular H_2_O_2_ and ClO^-^ release by neutrophils that is ineffective, however, to efficiently eradicate non-phagocytosed *H. pylori* (Ramarao et al., 2000; Allen et al., 2005). In addition to KatA, other antioxidant proteins such as KatB and AhpC are also present in the OMV proteome (Mullaney et al., 2009; Olofsson et al., 2010). However, we found that the catalase activity of OMVs is solely attributed to KatA accumulation since the ability to hydrolyse H_2_O_2_ was diminished in OMVs lacking KatA (**Figure 2B**). This could be due to the relatively low amount of KatB and AhpC compared to KatA in the *H. pylori* OMVs (Mullaney et al., 2009; Olofsson et al., 2010). Importantly, the KatA-dependent catalase activity of *H. pylori* OMVs is highly conserved among different strains (**Figure 2B**), further suggesting OMVs as important antioxidant particles.

In this study, we employed the direct H_2_O_2_ and NaClO bactericidal assay as an *in vitro* extracellular ROS respiratory burst model. Of note, *H. pylori* KatA counteracts the oxidative damage of H_2_O_2_ and OCl^-^ via different mechanisms, which are through its catalase hydrolysis activity and oxidation of KatA methionine residues, respectively (Wang et al., 2006; Benoit and Maier, 2016). Our results demonstrated that *H. pylori* OMVs effectively neutralized ROS and rescued bacteria from lethal oxidative damage (**Figures 2C,D**).

The strategy to promote bacterial infection by virulence factor enrichment in OMVs has also been reported with other pathogens. *Bacteroides* spp. escapes from antibiotics by decorating their OMV surface with cephalosporinases (Stentz et al., 2015). *Aggregatibacter actinomycetemcomitans* utilizes OMVs enriched with leukotoxin to induce immune cell apoptosis (Bonnington and Kuehn, 2014). Our finding pioneered the idea of virulence factor enrichment in OMVs as a novel virulence mechanism of *H. pylori.* This is exemplified by KatA in OMVs that, in turn, contributes to the novel antioxidative role of *H. pylori* OMVs, and thus enhanced bacterial defense against host innate immune attacks. We speculate that, during infection in gastric mucosa, *H. pylori* releases OMVs enriched with KatA to decrease or depelete the surrounding extracellular ROS released from the oxidative burst of influxed PMNs. This will allow *H. pylori* to escape towards nearby infection sites with lower ROS, thereby facilitating bacterial survival and colonization. In conclusion, we have presented expanded insights on a novel potential virulence mechanism of *H. pylori* that provide additional knowledge regarding bacterial survival in a hostile PMN-rich environment.

## Materials and Methods

### Bacterial Strains and Growth Conditions

Bacterial strains and growth conditions are listed in **Table 1**.

**Table 1:** List of bacterial strains used in this study.

Bacterial strain^a^	Description^b^	Reference or source
*H. pylori* 18943 wt	Wild type. Clinical isolate from a gastric antrum biopsy.	Culture Collection, University of Göteborg, Sweden (CCUG)
*H. pylori* CCUG18943Δ*katA*	Km^R^. Isogenic *katA* deletion mutant of CCUG18943 was constructed by replacement of *katA* with *nptI*. The strain is devoid of KatA expression.	Richter et al., 2016
*H. pylori* P12 wt	Wild type. Clinical isolate from a duodenal ulcer patient.	Schmitt and Haas, 1994
*H. pylori* P12Δ*katA*	Cm^R^. Isogenic *katA* deletion mutant of P12 by *cat* replacement. The strain is devoid of KatA expression.	This study

^*a*^*Helicobacter pylori* was grown on chocolate agar or in Brucella broth (Sigma-Aldrich, St. Louis, MO, United States) supplemented with 10% horse serum and 1% Vitox (Oxoid, Hants, United Kingdom) in microaerobic environment at 37°C. Liquid cultures were incubated at 200 rpm. ^*b*^Concentrations of antibiotics used: 30 μg/ml Kan; 20 μg/ml cm.

### Construction of *H. pylori* P12 *ΔkatA* Strain

*Kata* gene (Genbank Accession Number: CP001217; encodes KatA) deletion was performed as previously described (Richter et al., 2016). A linear katA-knockout construct containing *cat* (AY219687.1) was inserted between upstream and downstream flanking regions of the *katA* gene. Upstream flank was amplified from P12 gDNA using primers pair kat1F (5′-TCCCTTGAGCTGGTTGGCAATA-3′) and kat1R (5′-CTTAGCACTTGAGCCTAGAAGAGGCTGAGTACAGCATTG-3′). Primers kat2F (5′-CAATGGTGCCATGAATGGCAAAACCTCTTGGGTCTTTAC-3′) and kat2R (5′-CACCACAAGTAATTGGCCTAGTGTC-3′) were used to amplify downstream flank. Chloramphenicol resistance cassette was amplified from the gDNA of genomic DNA from strain J99sabB::cam using camF (5′-CAATGCTGTACTCAGCCTCTTC TAGGCTCAAGTGCTAAG-3′) and camR (5′-CGGTAAGAGACCCAAGAGGTT TTGCCATTCATGGCACCATTG-3′) primers. The construct containing a chloramphenicol resistance cassette and flanking regions was created by overlap extension PCR. For the overlap reaction of each PCR product were used as templates with primer pair kat1F and kat2R. All of PCR reactions were carried by GoTaq polymerase (Promega) or Phusion Hot start DNA polymerase (Thermo Scientific), and MJPTC-200 thermal cycler (MJ Research). The overlap PCR product was purified by E.Z.N.A Cycle Pure or Gel Extraction kits (OMEGA Bio-Tek, Norcross, GA, United States), prior to transformed into P12 wt. The mutant was verified by sequencing (Eurofin MWG, Ebersberg, Germany).

### OM and OMVs Preparation

To isolate OMVs, culture supernatants were concentrated using Vivaflow200 (Sartorius, Goettingen, Germany) and centrifuged at 165,000 ×*g* (Olofsson et al., 2010). Pellets were separated by Histodenz (20–50%), and centrifugation at 200,000 ×*g* and resuspended in phosphate buffered saline (PBS). OM was prepared from bacteria as described (Voss et al., 2014).

### Transmission Electron Microscopy (TEM)

The localization of KatA at the surface of intact bacteria and OMVs was determined by purified rabbit anti-KatA polyclonal antibodies (pAb) labeled with 5 nm colloidal thiocyanate gold followed by TEM using negative staining (Olofsson et al., 2010).

### Estimation of KatA Concentrations and Catalase Enzymatic Assays

Recombinant KatA (rKatA), OM, or OMVs sample were separated by SDS-PAGE followed by immunoblotting using anti-KatA pAb (**Supplementary Figure S1**) (Richter et al., 2016). Signal intensities generated from known amounts of rKatA were included as a standard curve for KatA estimation. Analysis was done by Image Lab software (Bio-Rad, Copenhagen, Denmark).

Bacterial lysates or OMVs were incubated with 1 mM H_2_O_2_ in catalase buffer (50 mM Tris pH 7.4, 0.1% TritonX-100) for 30 min at room temperature. The reaction was terminated with 50 mM sodium azide, and residual H_2_O_2_ was detected by OxiRed^TM^ (Biovision, Milpitas, CA, United States) and horseradish peroxidase (Thermoscientific, Waltham, MA, United States) mixture. Plates were read at 570 nm on a FLUOstar Omega microplate reader (BMG Labtech, Ortenberg, Germany).

### H_2_O_2_ Bactericidal Assay

H_2_O_2_ (1 mM) was preincubated with 40 μg/ml of OMV for 1 h at 37°C. Bacteria were resuspended in Brucella broth to an OD_600_ of 0.1, and added to the OMV-treated H_2_O_2_. Mixtures were incubated for 3 h at 37°C, and plated on chocolate agar for 5 days at 37°C to enumerate the bacterial survival based on colony forming units (CFU). Control experiments were performed as described above by using only H_2_O_2_ without OMVs.

### Hypochlorous Acid-Based Disk Diffusion Sensitivity Assay

A sterilized filter paper (5.4 mm in diameter) was saturated with 20 μl of 5% NaClO that had been pre-incubated for 3 h with OMVs. Bacterial colonies were resuspended in PBS and evenly spread on chocolate agar. Filter papers were placed on top of the agar, and plates were incubated at 37°C for 3 days. The diameter of inhibition zones was measured.

### Statistical Analysis

Graph-Pad Prism^®^ 7.0 (La Jolla, CA, United States) was used, and differences between groups or samples were considered statistically significant at *p* < 0.05.

## Author Contributions

AA, SL, Y-CS, and KR designed the study. AV, IO, MB, MA-K, and SL did experiments. KR, SL, and Y-CS wrote the manuscript. All authors have read and approved the submitted version.

## Conflict of Interest Statement

The authors declare that the research was conducted in the absence of any commercial or financial relationships that could be construed as a potential conflict of interest.
