# Supplementary material for: Helicobacter pylori Outer Membrane Vesicles Protect the Pathogen From Reactive Oxygen Species of the Respiratory Burst
Source: Front Microbiol. 2018 Sep 7;9:1837. doi: 10.3389/fmicb.2018.01837 (PMC6137165; doi:10.3389/fmicb.2018.01837)
Supplement: Supplementary file 1 [file Data_Sheet_1.docx]

Supplementary Material

*Helicobacter pylori* outer membrane vesicles protect the pathogen from reactive oxygen species of the respiratory burst

**Sujinna Lekmeechai^†^, Yu-Ching Su^†^, Marta Brant, Maria Alvarado-Kristensson, Anna Vallström, Ikenna Obi, Anna Arnqvist and Kristian Riesbeck^*^**

^†^*These a*uthors contributed equally to this manuscript

*** Correspondence:** Dr. Kristian Riesbeck: kristian.riesbeck@med.lu.se


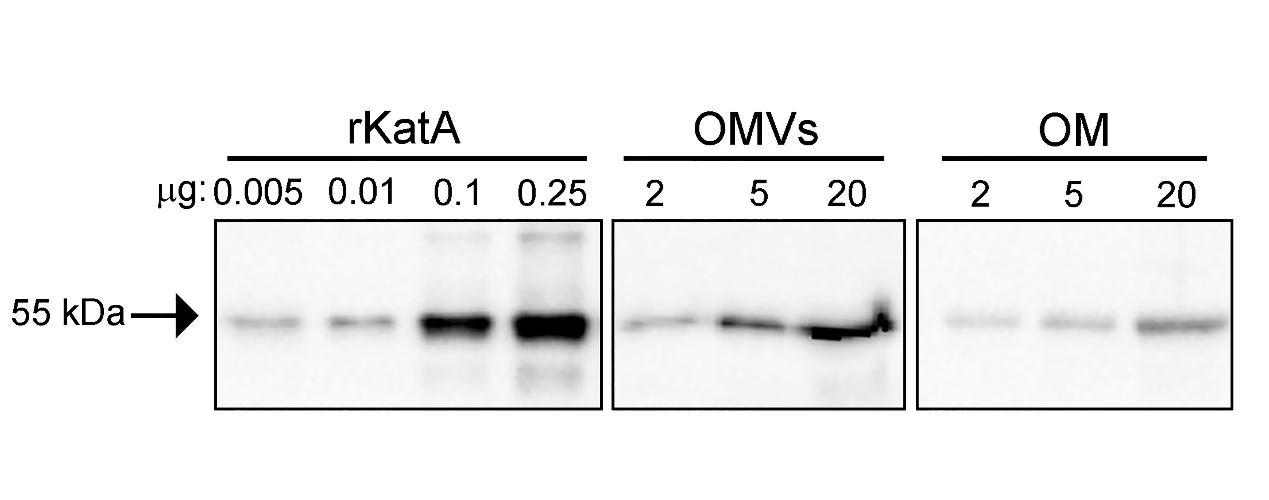


**Figure S1: Western blots for estimation of KatA concentrations in OMVs and OM derived from *H. pylori* 18943.** In order to estimate and compare the quantity of KatA in OM and OMVs, analyses with Western blots were performed. Purified recombinant KatA (0.005-0.25 μg/lane) (Richter et al., 2016), OM fraction (2-20 μg/lane), and OMVs (2-20 μg/lane) were separated by 12 % SDS-PAGE prior to transfer to PVDF membranes (0.4 μm pore size). KatA was detected with rabbit anti-KatA pAb (diluted 1:2,000 in 5% skimmed milk/PBS) (Richter et al., 2016) and a rabbit HRP-conjugated anti-mouse pAb (1:1,000 in 5% skimmed milk/PBS) (P016; Dako, Glostrup, Denmark). Signals were developed with ECL (Thermoscientific, Rockford, IL) and documented on a ChemiDoc^TM^ (Bio-Rad, Hercules, CA). After separation, signal intensity of each sample was measured, and KatA concentrations present in samples were determined using Image Lab Software version 4.0 (BioRad). A standard curve based upon recombinant KatA was used to estimate the amounts of KatA detected in OM and OMVs fractions.

**Reference**

1. Richter, C., Mukherjee, O., Ermert, D., Singh, B., Su, Y.-C., Agarwal, V., *et al*. (2016). Moonlighting of *Helicobacter pylori* catalase protects against complement-mediated killing by utilising the host molecule vitronectin. *Sci Rep*. 6, 24391. doi:10.1038/srep24391.
